# Post-procedural prostatic artery pseudoaneurysm causing severe hematuria treated with transcatheter embolization

**DOI:** 10.1016/j.radcr.2026.02.059

**Published:** 2026-03-23

**Authors:** Sean Lee, Karthik Rayasam, Lucas Struycken, Salil Kalarn, Jad El Bulbul, Ahmet Gunkan, Erol Bozdogan

**Affiliations:** Department of Radiology and Imaging Sciences, The University of Arizona College of Medicine, Tucson, Arizona, USA

**Keywords:** Prostatic artery pseudoaneurysm, Hematuria, Prostatic artery embolization, Interventional radiology

## Abstract

Prostatic artery pseudoaneurysm is an exceptionally rare vascular complication and an uncommon cause of gross hematuria, typically occurring after urologic instrumentation or surgical procedures such as transurethral resection of the prostate (TURP). We describe the case of a 73-year-old male with a history of TURP and recent lower urinary tract instrumentation who presented with recurrent gross hematuria and symptomatic anemia. Computed tomography angiography (CTA) of the abdomen and pelvis demonstrated a 1.1 × 0.6 cm pseudoaneurysm arising from the distal left prostatic artery adjacent to the prostatic apex. Given persistent bleeding despite conservative measures, the patient underwent image-guided transcatheter embolization using a microcatheter system and Gelfoam slurry, achieving complete resolution of hematuria without recurrence. This case demonstrates the diagnostic value of CTA in identifying rare vascular causes of hematuria and highlights targeted transcatheter embolization as an effective, minimally invasive definitive treatment for post-procedural prostatic artery pseudoaneurysm.

## Introduction

Massive hematuria can arise from a broad spectrum of etiologies, ranging from infection and malignancy to vascular injury. Pseudoaneurysm of the prostatic artery is an exceptionally uncommon cause, often associated with prior prostate interventions such as biopsy, TURP, or catheter-related trauma [1,2]. These vascular lesions can lead to life-threatening hemorrhage if not promptly recognized and managed.

CTA plays a pivotal role in identifying pseudoaneurysms and differentiating them from other causes of active extravasation or hematoma. While open surgical management has historically been employed, endovascular techniques such as prostatic artery embolization (PAE) now provide a safe and effective minimally invasive alternative for hemostasis and definitive treatment.

We describe a case of post-TURP prostatic artery pseudoaneurysm in an elderly male presenting with recurrent hematuria, successfully diagnosed by CTA and treated with image-guided embolization. This case highlights the complementary diagnostic and therapeutic roles of cross-sectional imaging and interventional radiology in managing rare vascular causes of urologic bleeding [3].

## Case report

A 73-year-old male with a remote history of TURP for benign prostatic hyperplasia (BPH) presented with recurrent gross hematuria and suprapubic discomfort. Twelve days prior to presentation, the patient underwent incision of a bladder neck contracture and urethral scar revision (September 5, 2025), with Foley catheter placement. Dual antiplatelet therapy (aspirin and ticagrelor) was resumed 48 hours after the procedure for a recent percutaneous coronary intervention.

Within 72 hours of restarting antiplatelet therapy**,** the patient developed worsening gross hematuria with clot formation and urinary retention. Despite continuous bladder irrigation and catheter management, hemoglobin declined to 8.2 g/dL, necessitating transfusion and prompting further diagnostic evaluation.

### CT findings

**Fig. 1:**
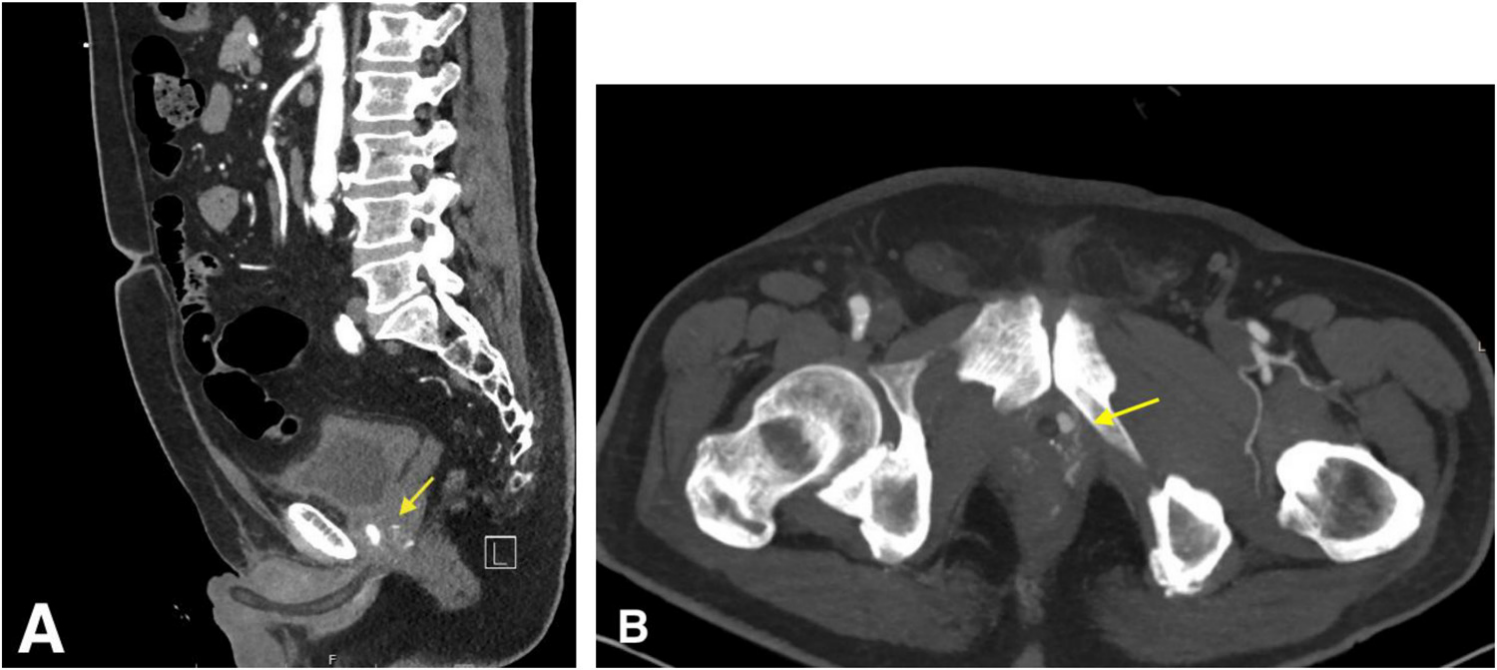
CTA of the pelvis demonstrates a left prostatic artery pseudoaneurysm. (A) Sagittal contrast-enhanced CT angiography demonstrates a well-defined, contrast-opacified pseudoaneurysm (arrow) at the left prostatic apex, along the lateral margin of the prostatic urethra. (B) Axial CT angiography again shows the pseudoaneurysm (arrow) adjacent to the Foley catheter, measuring 1.1 × 0.6 cm, with increased arterial enhancement consistent with prostatic arterial supply.

Multiphase contrast-enhanced computed tomography angiogram (CTA) of the abdomen and pelvis, including non-contrast, arterial phase, and delayed phase acquisitions to evaluate for active hemorrhage and vascular injury. Arterial phase images demonstrated a 1.1 × 0.6 cm contrast-opacified pseudoaneurysm arising from the distal left prostatic artery at the left prostatic apex adjacent to the prostatic urethra (Figs. 1A and B). No active contrast extravasation was seen.

Additional findings included intravesical clot burden without organized perivesical hematoma**,** no pelvic hematoma**,** hydronephrosis**,** or perivesical collection. The abdominal aorta, iliac, and mesenteric arteries were patent without aneurysm or flow-limiting stenosis. No additional sites of active hemorrhage were identified.

### Interventional procedure

**Fig. 2:**
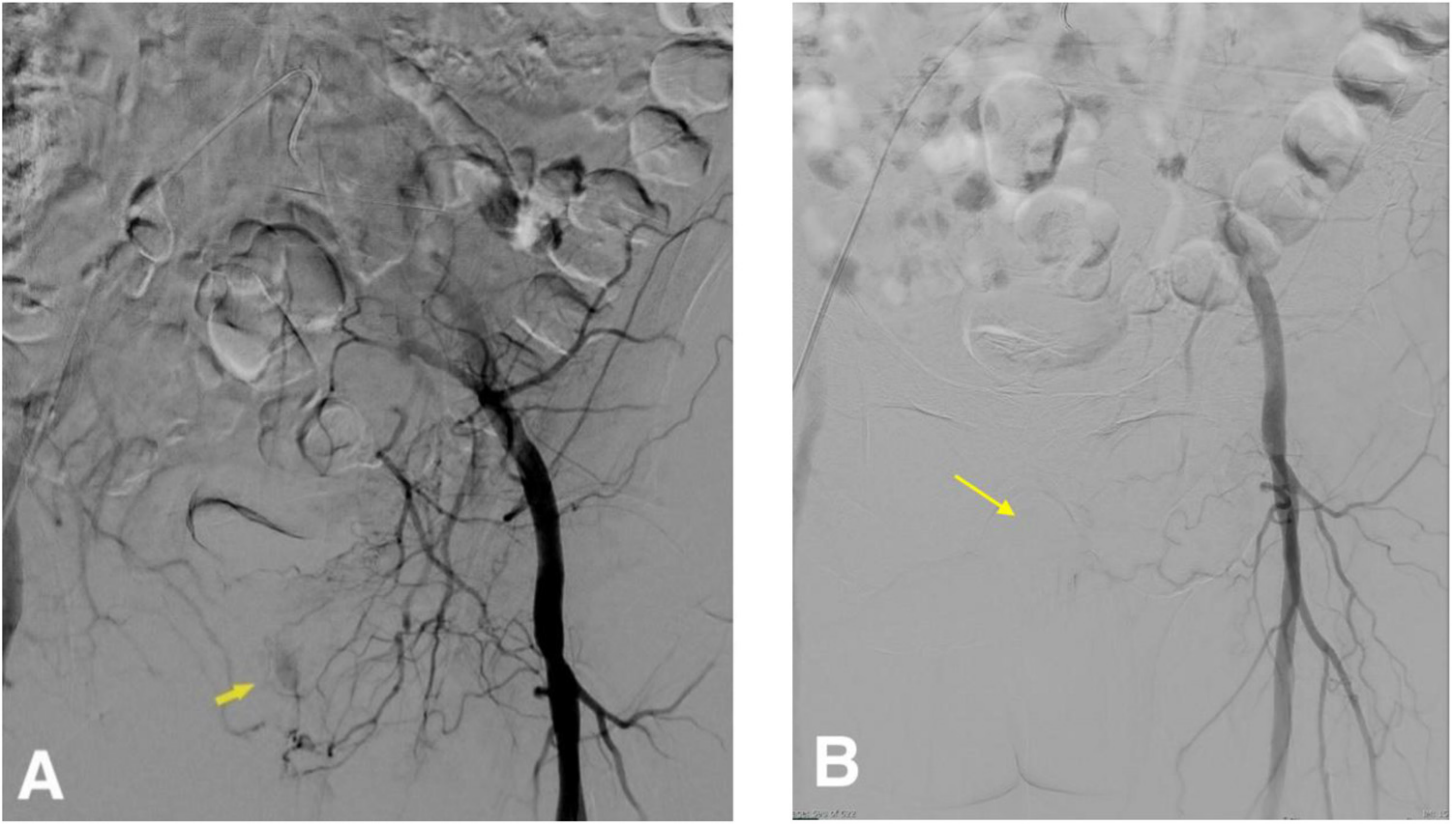
Digital subtraction angiography before and after embolization of the left prostatic artery pseudoaneurysm. (A) Selective internal pudendal artery angiogram demonstrates opacification of the distal left prostatic artery with direct filling of the pseudoaneurysm (arrow). (B) Post-embolization angiogram following Gelfoam slurry injection shows complete occlusion of the feeding artery and no further filling of the pseudoaneurysm (arrow)., confirming technical success.

The patient underwent image-guided transcatheter embolization of a left prostatic artery pseudoaneurysm under moderate conscious sedation. Right common femoral arterial access was obtained using ultrasound guidance and the Seldinger technique, followed by placement of a 5-French sheath. Sequential angiography of the left iliac system demonstrated conventional pelvic arterial anatomy with mild atherosclerosis and a pseudoaneurysm arising from the distal left prostatic artery, originating from the internal pudendal branch of the anterior division of the internal iliac artery (Fig. 2A).

Superselective catheterization of the left prostatic artery was achieved using a 2.0 Fr TruSelect microcatheter and Aristotle microwire system. Embolization was performed with Gelfoam slurry (approximately 3 mL diluted in contrast/saline mixture) under continuous fluoroscopic monitoring until angiographic endpoint of complete pseudoaneurysm exclusion with distal flow stasis was achieved.

A temporary embolic agent was selected to reduce the risk of permanent ischemic complications in adjacent prostatic and pelvic tissues, given the distal location and superselective catheterization. Risk of recanalization was mitigated by achieving complete pseudoaneurysm exclusion, dense distal stasis, and post-embolization angiographic confirmation of flow cessation.

Post-embolization angiography confirmed complete exclusion of the pseudoaneurysm with preservation of adjacent pelvic branches and no evidence of reflux or non-target embolization (Fig. 2B).

### Post-intervention clinical course

Hematuria resolved within 24 hours of embolization, allowing discontinuation of bladder irrigation. The patient received a total of 2 units of packed red blood cells during hospitalization. Hemoglobin at discharge was 9.8 g/dL.

**Fig. 3:**
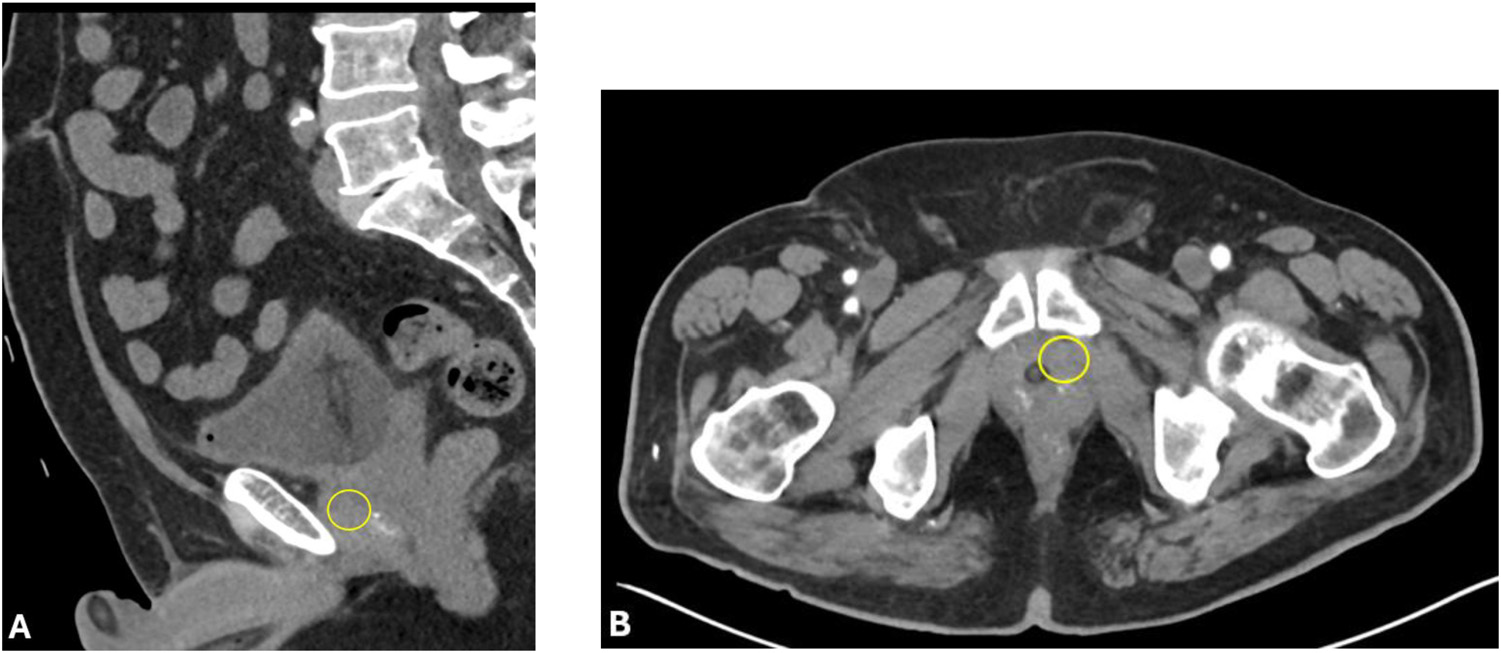
Post-embolization CT angiography demonstrating resolution of pseudoaneurysm. Post-embolization sagittal (A) and axial (B) CT angiography show complete interval resolution of the previously visualized pseudoaneurysm; the yellow circles mark the prior site of the lesion, which is no longer seen following successful embolization.

No recurrent bleeding occurred during hospitalization or outpatient follow-up**.** A follow-up CTA performed 7 days post-embolization demonstrated complete interval resolution of the pseudoaneurysm without recanalization (Figs. 3A and B). The patient remained clinically stable at 30-day outpatient follow-up with no recurrence of hematuria (Table 1).

**Table 1:** Overview of hospital course and key clinical events.

Time point	Key decision/finding	Action taken	Outcome
Presentation (day-1)	Gross hematuria with acute anemia (Hb 8.2 g/dL) and clot retention.	Initiated continuous bladder irrigation; transfused 2 units PRBCs; urology consultation.	Persistent hematuria prompting further diagnostic evaluation.
Diagnostic imaging (day-2)	CTA abdomen/pelvis identified a 1.1 × 0.6 cm pseudoaneurysm at the left prostatic apex arising from the distal prostatic artery.	Decision for endovascular intervention.	Target lesion identified for definitive therapy.
Definitive treatment (day-2)	Angiographic confirmation of culprit prostatic arterial pseudoaneurysm.	Image-guided prostatic artery embolization via right femoral access using Gelfoam slurry.	Completion angiography demonstrated complete occlusion with immediate improvement in hematuria.
Post‑procedure Course (day-3)	No recurrent bleeding; stable hemoglobin.	Discontinued continuous bladder irrigation; Foley catheter maintained.	Urine remained clear; no further transfusion required.
Discharge (day-5)	Clinical stability following embolization.	Routine post‑embolization observation; outpatient urology follow‑up arranged.	Discharged home with Foley catheter. Final hemoglobin at discharge: 9.8 g/dL.

## Discussion

Pseudoaneurysm formation of the prostatic artery represents an exceedingly rare vascular complication, originating from branches of the internal iliac artery, which itself accounts for only 0.3%-0.4% of all intra-abdominal aneurysms**.** When present, it may result in severe hematuria, clot retention, and hemodynamic compromise [4]**.** Most literature on PAE focuses on relief of lower urinary tract symptoms in BPH, but the same technical principles apply for hemorrhagic presentations [5]. Cross-sectional imaging, especially CTA, is critical for rapid identification and localization of the bleeding source, as it not only distinguishes pseudoaneurysms from active extravasation or nonvascular causes but also guides subsequent angiographic management. In this case, CTA precisely demonstrated a 1.1 × 0.6 cm pseudoaneurysm at the left prostatic apex, prompting immediate referral for embolization.

Endovascular management has become the mainstay for treating vascular complications of the prostate [3,5]. Retrospective studies have shown that transcatheter arterial embolization achieves technical success rates near 90% with initial bleeding control in over 80% of patients, establishing it as a safe and effective alternative to surgery for life-threatening urologic hemorrhage [5]. Similarly, a large cohort study demonstrated complete resolution of hematuria within three months following PAE in patients with BPH–related bleeding [6], while other series have shown durable hemostasis with low complication rates [7].

In this patient, superselective embolization using Gelfoam slurry achieved complete occlusion of the pseudoaneurysm without non-target embolization or recurrence. This case reinforces that minimally invasive endovascular management can be both diagnostic and curative. Early use of CTA to identify the bleeding source, followed by targeted PAE, represents an ideal diagnostic–therapeutic continuum for prostatic vascular injury. As awareness of this complication grows, PAE should be considered a first-line option in patients presenting with hematuria of vascular origin following prostate intervention.

## Conclusion

Prostatic artery pseudoaneurysm is a rare but serious vascular cause of hematuria, most often related to prior prostate surgery or instrumentation. This case illustrates the pivotal role of CTA in rapidly identifying the bleeding source and guiding targeted therapy. PAE provided a safe, effective, and minimally invasive means of achieving definitive hemostasis, avoiding the need for surgical intervention. Early recognition of this vascular complication and timely collaboration between urology and interventional radiology teams are critical to optimize outcomes and prevent recurrence.

### Learning points

• Prostatic artery pseudoaneurysm is a rare etiology of hematuria, typically occurring after TURP or instrumentation.
• CTA is the preferred imaging modality for noninvasive localization of the bleeding source.
• PAE offers a safe, minimally invasive treatment option that provides both diagnostic confirmation and therapeutic control.
• Early multidisciplinary involvement of urology and interventional radiology improves diagnostic accuracy and patient outcomes.
• Prompt recognition of vascular causes of hematuria is critical, especially in patients on dual antiplatelet or anticoagulant therapy, to prevent hemodynamic deterioration.

## Author contributions

All authors contributed to the conception, drafting, and review of this case report.

## Patient consent

Written informed consent for the publication of this case report was obtained from the patient.

## References

[1] Gonzalez-Araiza G., Haddad L., Patel S., Karageorgiou J. (2019). Percutaneous Embolization of a Postsurgical Prostatic Artery Pseudoaneurysm and Arteriovenous Fistula. Journal of Vascular and Interventional Radiology.

[2] Bonne L., Gillardin P., De Wever L., Vanhoutte E., Joniau S., Oyen R. (2017). Endovascular Management of Severe Arterial Haemorrhage After Radical Prostatectomy: A Case Series. Cardiovasc Intervent Radiol.

[3] Amouyal G., Tournier L., De Margerie-Mellon C., Pachev A., Assouline J., Bouda D. (2022). Safety Profile of Ambulatory Prostatic Artery Embolization after a Significant Learning Curve: Update on Adverse Events. Journal of Personalized Medicine.

[4] Arya M.C., Kumar L., Mittal R., Kumar R., Baid M. (2016). Posttransurethral Resection of Prostate Recurrent Life Threatening Hematuria: A Rare Cause. Case Rep Urol.

[5] Delgal A., Cercueil J., Koutlidis N., Michel F., Kermarrec I., Mourey E. (2010). Outcome of transcatheter arterial embolization for bladder and prostate hemorrhage. J Urol.

[6] Hijazi B.A., Shi H., Liu S., Wei T., Alqurashi T., Sabri Z. (2023). Safety and efficacy of prostatic artery embolization in patients with hematuria due to benign prostate hyperplasia. African Journal of Urology.

[7] Carnevale F.C., Soares G., Moreira de Assis A., Moreira A., Harward S. (2017). Anatomical Variants in Prostate Artery Embolization: A Pictorial Essay. Cardiovasc Intervent Radiol.

